# Enhanced Network in Corticospinal Tracts after Infused Mesenchymal Stem Cells in Spinal Cord Injury

**DOI:** 10.1089/neu.2022.0106

**Published:** 2022-11-30

**Authors:** Ryosuke Hirota, Masanori Sasaki, Yuko Kataoka-Sasaki, Tsutomu Oshigiri, Kota Kurihara, Ryunosuke Fukushi, Shinichi Oka, Ryo Ukai, Mitsunori Yoshimoto, Jeffery D. Kocsis, Toshihiko Yamashita, Osamu Honmou

**Affiliations:** ^1^ Department of Neural Regenerative Medicine, Research Institute for Frontier Medicine, and Sapporo Medical University School of Medicine, Sapporo, Japan.; ^2^ Department of Orthopaedic Surgery, Sapporo Medical University School of Medicine, Sapporo, Japan.; ^3^ Department of Neurology, and Yale University School of Medicine, New Haven, Connecticut, USA.; ^4^ Department of Neuroscience, Yale University School of Medicine, New Haven, Connecticut, USA.; ^5^ Center for Neuroscience and Regeneration Research, VA Connecticut Healthcare System, West Haven, Connecticut, USA.

**Keywords:** axon tracing, mesenchymal stem cell, spinal cord injury

## Abstract

Although limited spontaneous recovery occurs after spinal cord injury (SCI), current knowledge reveals that multiple forms of axon growth in spared axons can lead to circuit reorganization and a detour or relay pathways. This hypothesis has been derived mainly from studies of the corticospinal tract (CST), which is the primary descending motor pathway in mammals. The major CST is the dorsal CST (dCST), being the major projection from cortex to spinal cord. Two other components often called “minor” pathways are the ventral and the dorsal lateral CSTs, which may play an important role in spontaneous recovery. Intravenous infusion of mesenchymal stem cells (MSCs) provides functional improvement after SCI with an enhancement of axonal sprouting of CSTs. Detailed morphological changes of CST pathways, however, have not been fully elucidated. The primary objective was to evaluate detailed changes in descending CST projections in SCI after MSC infusion. The MSCs were infused intravenously one day after SCI. A combination of adeno-associated viral vector (AAV), which is an anterograde and non-transsynaptic axonal tracer, was injected 14 days after SCI induction. The AAV with advanced tissue clearing techniques were used to visualize the distribution pattern and high-resolution features of the individual axons coursing from above to below the lesion. The results demonstrated increased observable axonal connections between the dCST and axons in the lateral funiculus, both rostral and caudal to the lesion core, and an increase in observable axons in the dCST below the lesion. This increased axonal network could contribute to functional recovery by providing greater input to the spinal cord below the lesion.

## Introduction

Spontaneous recovery of motor function, although limited, is observed after spinal cord injury (SCI).^1^ This functional improvement occurs gradually and can be exhibited for several months^2^ and even years^3^ after SCI. Although the underlying mechanisms evoking endogenous recovery are still unclear, current knowledge reveals that multiple forms of axon growth in spared axons can lead to circuit reorganization and a detour or relay pathways that may contribute to spontaneous recovery.^4,5^

This hypothesis has been derived mainly from studies of the corticospinal tract (CST), which is the major descending motor pathway in all mammalian species.^6^ The CST has multiple components with the dorsal CST (dCST) being the major projection from the cortex to the spinal cord; it contains approximately 90% to 95% of the descending CST axons. In rats, dCST axons cross in the decussation of the medullary pyramids and descend deep in the dorsal funiculus of the spinal cord.

Two other pathways are the ventral CST (vCST) and dorsal lateral corticospinal tract (dlCST). The vCST does not cross in the pyramids, but descends ipsilateral in the ventromedial aspect of the ventral funiculus (VF). The vCST axons, however, can cross locally in the spinal cord at the level of innervation. The dlCST travels in the dorsal portion of the lateral funiculus (LF) and is crossed. These well-known minor components of the CST are numerically small but may play an important role in the endogenous recovery of function.^7,8^

Intravenous infusion of mesenchymal stem cells (MSCs) is a promising therapy for SCI to provide functional improvement.^9–14^ Infused MSCs previously have been suggested to enhance axonal sprouting of CST fibers in SCI.^11,15^ The fine morphological features of CST pathways, including major and minor components in the injured spinal cord after MSC infusion, have not been well studied, however.

In this study, we investigated the differential pattern in distribution of the CST in the spinal cord with anterograde axonal tracing using an adeno-associated viral (AAV) vector after intravenous infusion of MSCs in a rat model of SCI. The combination of the AAV anterograde axonal tracer, which is non-transsynaptic, with advanced tissue clearing techniques allowed us to study the fine morphological features of the neural circuits formed with individual axons between major and minor CST projections in the spinal cord rostral and caudal to the lesion core and changes effectuated after intravenous infusion of MSCs in SCI.

## Methods

### Animals

All experiments were conducted in accordance with the institutional guidelines of Sapporo Medical University. The use of animals in this study was approved by the Animal Care and Use Committee and the Committee for Security of Recombinant DNA Experiments of Sapporo Medical University.

### Preparation of MSCs from rat bone marrow

The MSC preparation and culture were conducted based on our previous studies.^16^ Briefly, bone marrow, obtained from femoral bones in adult (6–8 weeks old) Sprague-Dawley (SD) rats, was diluted to 15 mL with Dulbecco modified Eagle medium (DMEM) (Sigma, St. Louis, MO) supplemented with 10% heat-inactivated fetal bovine serum (Thermo Fisher Scientific Inc., Waltham, MA), 2 mM l-glutamine (Sigma), 100 U/mL penicillin, and 0.1 mg/mL streptomycin (Thermo Fisher Scientific Inc.) and incubated for three days at 37 °C in a humidified atmosphere containing 5% CO_2_. When cultures almost reached confluence, the adherent cells were detached with a trypsin-ethylenediaminetetraacetic acid solution (Sigma) and subcultured at 1 × 10^4^ cells/mL of medium. After three passages, the MSCs were used in the present study. A previous phenotypic analysis of the surface antigens revealed cluster of differentiation (CD) 45^-^, CD73^+^, CD90^+^, and CD106^-^ on MSCs.^17,18^

### SCI model

Contusive SCI was performed as described previously.^12^ Briefly, adult (7-week-old) male SD rats (250–300 g) were anesthetized with an intraperitoneal (IP) injection of ketamine (90 mg/kg, IP) and xylazine (4 mg/kg, IP). After skin incision, the T9 vertebra was stabilized, a laminectomy was performed at the T9–10 level of the spinal cord, and a 150-kdyn contusion was delivered to the spinal cord using an Infinite Horizons impactor (Precision Systems and Instrumentation, LLC, Lexington, KY). Appropriate post-operative care was provided for all animals, including twice-daily manual bladder expression for up to 14 days. The rats were housed in an atmosphere of 50% humidity at a temperature of 24 ± 2°C.

### Behavioral testing

Open field locomotor function was assessed by a tester blinded to the treatment using the Basso, Beattie, and Bresnahan (BBB) locomotor rating scale.^19^ Intact (*n* = 10) and SCI rats (*n* = 20) were scored two days before SCI induction, and at two-day intervals thereafter until sacrifice at eight weeks post-SCI induction.

### Experimental protocol

Only rats with BBB scores that displayed zero points one day after SCI induction were included in this study. The SCI rats with BBB scores of zero points were randomized and received a single intravenous infusion of MSCs at 1.0 × 10^6^ cells in 1.0 mL of fresh DMEM (*n* = 10) or vehicle (1.0 mL fresh DMEM alone) (*n* = 10) via the femoral vein one day after SCI induction. All rats were injected daily with cyclosporine A (10 mg/kg, IP).^10,11,20^ Age-matched intact rats were used as intact controls (*n* = 10). At day 14 after SCI induction, AAV virus was injected into both SCI animals and age-matched intact animals^21–26^ (see Neuroanatomical tracing). Eight weeks after SCI induction, histological analysis was performed.

### Neuroanatomical tracing

Green Fluorescent Protein (GFP)-encoding AAVs with a chicken beta-actin (CAG) promoter and tdTomato-encoding AAVs with a CAG promoter (AAV-8-CAG-GFP/tdTomato) were purchased from Vector Biolabs (Malvern, PA). At day 14 after SCI induction, the rats were placed on a stereotaxic frame under anesthesia induced by an IP injection of ketamine (75 mg/kg) and xylazine (10 mg/kg).

Fourteen days post-SCI, a craniotomy was performed to expose the sensorimotor cortex. The GFP-encoding AAV was injected into the right hemisphere. The tdTomato-encoding AAV was injected into the left hemisphere. Five rats per group (five intact, five vehicle-SCI, five MSC-SCI) received both AAV-8-CAG-GFP/tdTomato. Five rats per group (five intact, five vehicle-SCI, five MSC-SCI) received both AAV-8-CAG-tdTomato.

For each hemisphere, six injections for cortex (AAVs; 4.0 × 10^10^ genome copy/μL, 0.5 μL per site) were performed at the following coordinates: 1.0 mm lateral; 1.5 mm, 1.0 mm depth; and -1.0 mm, 0 mm, 1.0 mm posterior to the bregma using a nanoliter-injector (World Precision Instrument Inc., Sarasota, FL) attached to a pulled glass pipette.^27^ The method used in this study allowed us to perform precise microdelivery of viral vectors to localized regions in the brain. The needle was left in place for 3 min before moving to the next site.

### Histological analysis

Six weeks after tracer injections,^28^ rats were perfused transcardially with cold phosphate-buffered saline (PBS) followed by 4% paraformaldehyde under deep anesthesia with an IP injection of ketamine (75 mg/kg) and xylazine (10 mg/kg). Spinal cords were dissected out and stored at −80°C until use. Sections were cut into 50-μm thickness using a cryostat (Sakura Seiki Co, Tokyo, Japan).

One section of each animal was selected and cut. Then, these sections were washed three times in PBS containing 0.1% Tween 20 (PBS-T) three times. The sections were examined using a confocal microscope (Zeiss LSM780 ELYRA S.1 system). Sections were viewed directly to assess GFP and tdTomato fluorescence. The intensities of GFP or tdTomato signals were quantified using ImageJ software bundled with Java 1.8.0_172 (National Institutes of Health [NIH], Bethesda MD).^27,29,30^

### Clearing

The spinal cord tissue was cleared according to a modified version of the advanced clear, unobstructed brain imaging cocktails and computational analysis (CUBIC) protocol.^31^ First, the samples were treated with half-diluted CUBIC-L (#T3740, Tokyo Chemical Industry Co., Ltd., Tokyo, Japan). The fixed samples were immersed in 10 mL of half-diluted CUBIC-L for 6 h at 37°C under low-speed rotation. The samples were then placed in 10 mL of 100% CUBIC-L solution for four days at 37°C with gentle shaking. The CUBIC-L was replaced every 2 days.

Then, to wash off the remaining CUBIC-L reagent, the samples were washed in 10 mL of PBS for 2 h at room temperature with gentle shaking. The samples were then immersed in 5 mL of CUBIC-R+ (for animals) (#T3741, Tokyo Chemical Industry, Co., Ltd.) diluted by half and treated for 24 h at room temperature. Finally, the samples were immersed in 4 mL of CUBIC-R+ and shaken gently at room temperature overnight. The next day, fresh reagents were replaced and incubated for another 24 h before imaging.

### Light sheet fluorescence microscopy (LSFM) of optically cleared samples

The LSFM of optically cleared rat spinal cords was performed with an Ultramicroscope II (Miltenyi Biotec, Germany), including an Olympus MVX10 zoom body (Olympus), and a scientific complementary metal–oxide–semiconductor camera (PCO, Germany) with a pixel size of 6.5 μm. Detection optics with an optical magnification range of 1.26 to 12.6 and an NA of 0.5 were used. For tdTomato and GFP excitation, a 561-nm and 488-nm diode laser was used, respectively. The emitted wavelengths were detected with specific detection filters: 620/60 nm for tdTomato and 525/50 nm for GFP. The optical zoom factor of the measurements varied from 1.26 to 8 and the light-sheet thickness ranged from 5 to 10 μm.

### Quantitative analysis

Images of sections labeled with tdTomato (*n* = 5/group), each at the C4-5 and L1-2 levels, were acquired using a Zeiss microscope. The distribution of axons was plotted using the ImageJ software (NIH). Their distributions were further calculated in squares, which divided the hemispinal cord image by 6 × 9, and heatmaps were generated using Graph-R software.^28^ The value in each square was assigned into 20 divisions ordered from high to low numbers with the squares in the highest division represented in red and the lowest in blue color.

### Statistics

All statistical analyses were performed using the Statistical Package for the Social Sciences 21 for Macintosh (IBM, Inc., IL). Groups were compared by one-way analysis of variance, and the Tukey-Kramer test was used for *post hoc* comparisons. Comparisons between two groups were performed using the Mann-Whitney *U test*. Data are expressed as mean ± standard error of the mean. Differences were considered statistically significant at *p* < 0.05.

### Data availability

The data that support the findings of this study are available from the corresponding author on reasonable request.

## Results

### Infused MSCs provide improved locomotor function

Open field locomotor function was assessed using the BBB behavioral score starting immediately before and three days after MSC or vehicle infusion to confirm injury equivalency across animals with SCI, as well as to determine whether infused MSCs affected overall locomotor ability. All animals demonstrated near-complete hindlimb paraplegia before infusion (1 day after SCI) and then exhibited a gradual improvement.

**FIG. 1.**
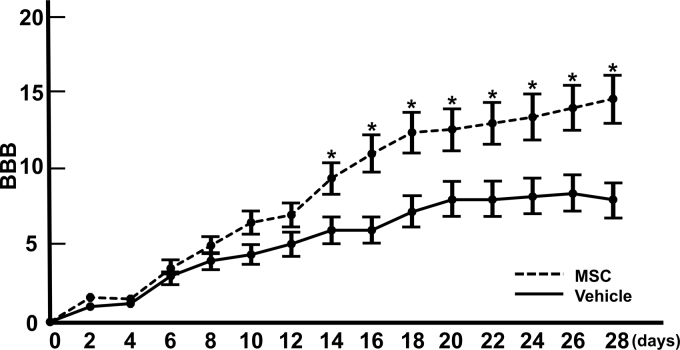
Behavioral analysis of locomotor function. Open field locomotor scores and Basso–Beattie–Bresnahan (BBB) score for each group were tested at 2, 4, 6, 8, 10, 12, 14, 16, 18, 20, 22, 24, 26, and 28 days after SCI. All animals showed a gradual improvement in hindlimb locomotor function during the four-week recovery period. Statistical analysis indicated that the BBB scores in the mesenchymal stem cell (MSC) transplantation groups were significantly higher than those in the vehicle group (medium alone) at 14, 16, 18, 20, 22, 24, 26, and 28 days after spinal cord injury. *p < 0.05.

The MSC-treated rats demonstrated markedly improved locomotor performance, as evidenced by higher BBB scores after MSC infusion, which increased throughout the 28-day study period, while the performance of the vehicle-infused rats did not improve after day 20. Scores on the BBB in the MSC-infused group were significantly higher than those of the vehicle-treated group at 14 days (9.11 ± 1.14 vs. 6.0 ± 0.89; *p* < 0.05), 16 days (10.96 ± 1.10 vs. 6.0 ± 0.89; *p* < 0.05), 18 days (12.22 ± 1.29 vs. 7.18 ± 0.96; *p* < 0.05), 20 days (12.66 ± 1.25 vs.7.94 ± 1.12; *p* < 0.05), 26 days (13.96 ± 1.26 vs. 8.22 ± 1.15; *p* < 0.05), and 28 days (14.64 ± 1.30 vs. 8.05 ± 0.98; *p* < 0.05) after infusion, respectively (Fig. 1).

The *p* values were determined using the Mann-Whitney *U* test. The BBB scores of intact animals were 21 during the study period. These results indicated that the infused MSCs provided therapeutic efficacy in a rat model of SCI. The SCI model system was used in the current study.

### Main component of the CST

**FIG. 2.**
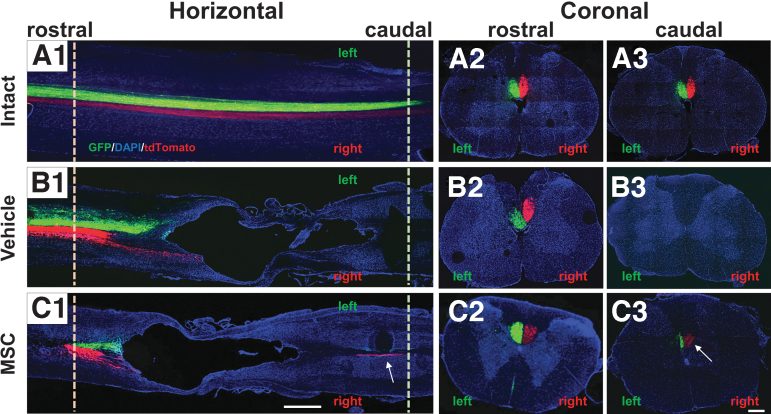
Confocal microscopic observation of spinal cords injected with adeno-associated viral vector-8-chicken beta-actin-green fluorescent protein (AAV-8-CAG-GFP—green) virus into the right cortex and AAV-8-CAG-tdTomato (red) into the left cortex. The horizontal spinal sections of the intact (**A1**), vehicle (**B1**), and mesenchymal stem cell (MSC) (**C1**) groups. The dashed line at the rostral area in horizontal sections (pale orange) corresponds to the coronal sections of intact (**A2**), vehicle (**B2**), and MSC (**C2**), respectively. The dashed line at the caudal area in horizontal sections (light green) corresponds to the coronal sections of intact (**A3**), vehicle (**B3**), and MSC (**C3**), respectively. Arrow in C1 and C3 shows the converged dorsal corticospinal tract (dCST) at the caudal to the lesion core only in the MSC group. Scale bars = 1 mm (A1, B1, C1) and 300 μm (A2, A3, B2, B3, C2, C3).

To visualize CST axons in the spinal cord, we used AAV anterograde tracers. The AAV-8-CAG-GFP virus was injected into the right cortex, and AAV-8-CAG-tdTomato was injected into the left cortex. Six weeks later, horizontal frozen sections obtained from intact rat spinal cord showed robust labeling of the bilateral CST axons in the dCSTs (left; GFP, right; tdTomato) (Fig. 2A1). Coronal sections in the intact spinal cord (T9–10) also display distinct dCST axons located at the base of the dorsal funiculus. These data are shown at longitudinal positions of approximately 5-mm rostral (Fig. 2A2) and 5-mm caudal (Fig. 2A3) to the approximated center of the targeted contusion injury site. These data demonstrate that the AAV tracing method used is robust and labels the main CST axons in the spinal cord.

In the vehicle-infused SCI rats, almost all of the fibers in the main CST were lost distal to the lesion site by eight weeks after the injury, and a substantial cavity formed in the lesion core (Fig.2 B1). Although the coronal section rostral to the lesion core exhibited distinct dCST axons in the dorsal funiculus (Fig. 2B2), similar to the intact animal, no dCST axons were present in the section caudal to the lesion core (Fig. 2B3).

Surprisingly, although less in number than in the intact spinal cord, distinct dCST axons were prominent caudal to the lesion core in the MSC-infused SCI rats (Fig. 2C1, Fig. 2C3, arrow). These CST axons, which converged deep in the dorsal funiculus to approximate the location of the dCST, were only observed in the MSC-treated animals. In coronal sections caudal to the lesion core (Fig. 2C3), distinct CSTs expressing GFP and tdTomato exist on the appropriate side of the DF—that is, the same laterality as rostral to the lesion core (Fig. 2C2), respectively. The arrow in the horizontal section in Fig. 2C1 corresponds to the arrow in the coronal section of Fig. 2C3.

### Distribution of CST axons

**FIG. 3.**
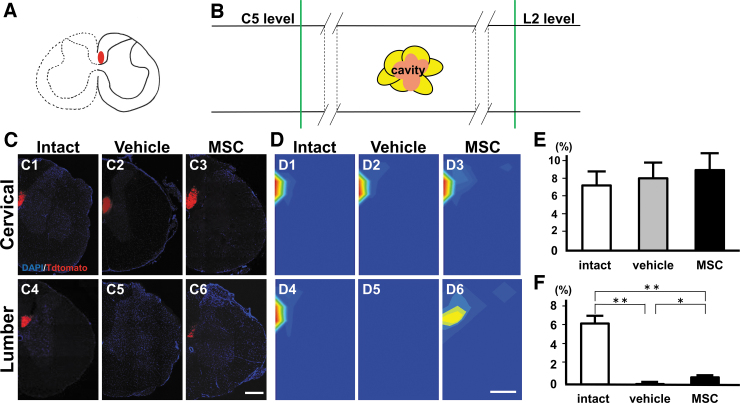
Distribution of the corticospinal tract (CST). (**A**) Schema of coronal spinal cord injected with adeno-associated viral vector-8-chicken beta-actin-green fluorescent protein-tdTomato (AAV-8-CAG-tdTomato—red). (**B**) Schema of the horizontal spinal cord showing the longitudinal levels of analyzed coronal sections (green lines) of CST rostral (C5 level) and caudal to the lesion (L2 level). Confocal images (**C**) of coronal spinal cord injected with AAV-8-CAG-tdTomato (red) into the left cortex at cervical spinal cord (intact, C1; vehicle, C2; mesenchymal stem cell [MSC], C3) and lumbar spinal cord (intact, C4; vehicle, C5; MSC, C6) counterstained with 4',6-diamidino-2-phenylindole (DAPI—blue). Axon distribution heatmaps (**D**) at cervical spinal cord (intact, D1; vehicle, D2; MSC, D3) and lumbar spinal cord (intact, D4; vehicle, D5; MSC, D6). Note that converged signals of the dorsal CST are observed in D6. Quantification heatmap analysis at cervical spinal cord (**E**) and lumbar spinal cord (**F**). Scale bars = 500 μm (B, C). *p < 0.05, **p < 0.01.

The schematic illustration in Fig. 3A shows the coronal spinal cord with AAV-8-CAG-tdTomato in the dCST (red), and Fig. 3B displays areas of CST axonal analysis rostral (cervical level five) and caudal to the lesion (lumbar level two); to quantify the distribution of CST axons obtained from confocal images in Fig. 3C, constructed regional density heatmap analyses were performed (Fig. 3D).

The heatmap of CST axons at the cervical level (Fig. 3D1–3D3) and lumbar level (Fig. 3D4–3D6) of the spinal cord shows the topographic distribution of AAV^+^ axons across experimental groups. As observed in Figure 2, tdTomato^+^ CST axons in the dCST were visible rostral to the lesion (Fig. 3C1) in intact and injured spinal cord animals in the vehicle (Fig. 3C2) and MSC (Fig. 3C3) groups. In intact animals, tdTomato^+^ CST axons in the dCST were prominent caudal to the lesion (Fig. 3), but there were no signals caudal to the injury in the vehicle group (Fig. 3C5). In the MSC group, however, CST signals caudal to the injury converged to the anticipated position of the dCST (deep in the dorsal funiculus) (Fig. 3C6).

Heatmap analysis indicated that signal intensity rostral to the lesion was elevated in all experimental groups (Fig. 3D1–D3). While signal intensity was elevated caudal to the lesion in the intact group (Fig. 3D4), however, no signal was detected caudal to the lesion in the vehicle group (Fig. 3D5). Distinct dCST signals were evident caudal to the lesion core in the MSC-infused SCI rats (Fig. 3D6), but with less signal intensity than in the intact spinal cord (Fig. 3D4). Graphs from the quantitative heatmap analysis demonstrated that there was no difference among the three groups rostral to the injury (cervical level) in CST fibers (Fig. 3E).

Heatmap signals of CST fibers at the lumbar level (caudal to the injury) in the intact group were greater than those in the vehicle group, which had no signal, and greater than the MSC group, which had a weaker signal (Fig. 3F), suggesting that infused MSCs facilitated the convergence of regenerated axons to the anatomical position of the dCST caudal to the lesion.

### Rostral dispersion and caudal convergence of CST after infused MSCs

**FIG. 4.**
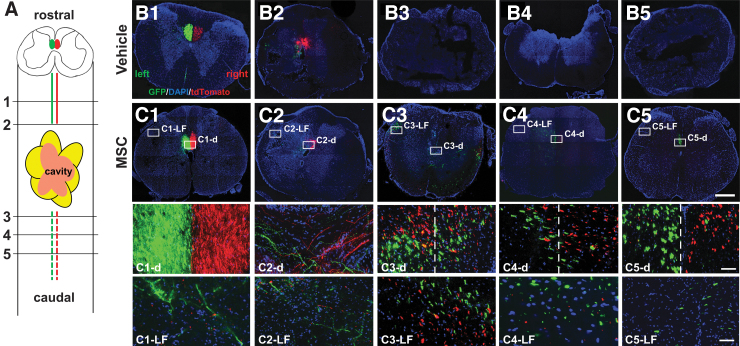
Rostral dispersion and caudal convergence of the corticospinal tract (CST) after infused vehicle and mesenchymal stem cells (MSCs). (**A**) Schema of the spinal cord with cavitation showing the areas of CST confocal analysis rostral (black lines; 1, 2) and caudal to the lesion (black lines; 3, 4, 5). Confocal images of coronal spinal cords injected with adeno-associated viral vector-8-chicken beta-actin-green fluorescent protein (AAV-8-CAG-GFP—green) virus into the right cortex and AAV-8-CAG-tdTomato (red) into the left cortex after infused vehicle (**B**) and MSCs (**C**). Boxed areas in Cn (*n* = 1, 2, 3, 4, 5) show the spinal cords (Cn-LF) in the lateral funiculus (LF) and a dorsal funiculus (Cn-d), respectively. The dashed lines in C3-d, C4-d, and C5-d indicate the midlines of the dorsal funiculus. Scale bars = 500 μm (B1 to B5, C1 to C5), 50 μm (C1-d to C5-d, C1-LF to C5-LF).

For a detailed comparative investigation in animals with SCI after vehicle (Fig. 4B) and MSC (Fig. 4C) infusions, closely spaced coronal frozen sections above and below the SCI site were examined. The schematic in Fig. 4A indicates the approximate levels of sections above (1, 2) and below (3, 4, 5) the lesion. Sections from vehicle-treated rats above and below the lesion are shown in Fig. 4B1, B2, and Fig. 4B3-B5, respectively. Sections above and below the lesion in the MSC-treated group are shown in Fig. 4C1, C2, and Fig. 4C3–C5, respectively.

Similar to Figure 3, distinct dCST axons were obvious in the closely spaced sections rostral to the lesion in both vehicle (Fig. 4B1, 4B2) and MSC (Fig. 4C1, 4C2) groups. No dCST axons caudal to the lesion in the vehicle group (Fig. 4B3–B5) were detected, even though distinct axons in the dCST were prominent caudal to the lesion core in the MSC-infused SCI rats (Fig. 4C3–4C5).

Rostral to the lesion site in the MSC group, distinct GFP^+^ and tdTomato^+^ dCST axons were located at the base of the DF with appropriate laterality above the lesion core (Fig. 4C1, 4C1-d, Fig. 4C2). The bottom two rows of Fig. 4 show the expansions of the boxes in C1–C5. Scattered GFP^+^ and tdTomato^+^ AAV axons were also observed dispersed in the LF above the lesion (Fig. 4C1-LF and Fig. 4C2-LF) from the DF (Fig. 4C2, Fig. 4C2-d). The LF and d in Fig. 4 refer to the LF and dorsal funiculus, respectively. Note that GFP^+^ and tdTomato^+^ AAV axons were more comingled closer (position 2) to the lesion core (Fig. 4C2-d and Fig. 4C2-LF).

Caudal to the lesion, both scattered GFP^+^ and tdTomato^+^ AAV axons converged to the base of the DF (position of the dCST in intact animals) in the MSC group (Fig. 4C3, Fig. 4C4), and the most distal section (position 5) was largely appropriately lateralized (Fig. 4C5). In regions just caudal to the lesion core displayed in Fig. 4C3 and C4, the mixture of GFP^+^ and tdTomato^+^ AAV axons was visible on both sides of the DF (Fig.4 C3-d, Fig. 4C4-d [dashed lines indicate the middle of the dorsal funiculus]). Mixed left and right axons were also observed in the LF (Fig. 4C3-LF, Fig. 4C4-LF).

The converged GFP^+^ and tdTomato^+^ AAV axons, however, were gradually positioned to their appropriate lateralization (Fig. 4C5) as in sections rostral to the lesion (Fig. 4C1) and in the intact group caudal to the lesion (see Fig. 2A3). This is clearly shown in the boxed area (Fig. 4C5-d) in Fig. 4C5. Note that GFP^+^ AAV axons were predominantly observed caudal to the lesion in the LF (Fig. 4C5-LF).

### Distribution of CST axons in the lesion core

**FIG. 5.**
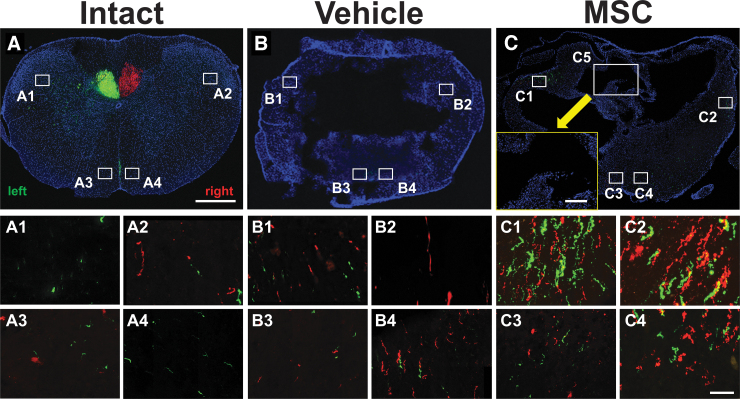
Distribution of corticospinal tract (CST) axons in the lesion core. Confocal images of coronal spinal cord in intact (**A**), vehicle (**B**), and mesenchymal stem cell (MSC) (**C**) groups injected with adeno-associated viral vector-8-chicken beta-actin-tdTomato (AAV-8-CAG-tdTomato—red) into the left cortex at the lesion core counterstained with 4',6-diamidino-2-phenylindole (DAPI—blue). Note the higher magnification (yellow inset box in C) of the dorsal funiculus (C5) showing complete abolishment of the area of the dorsal CST after spinal cord injury. Boxed areas 1 and 2 refer to the lateral funiculus, and areas 3 and 4 refer to the ventral funiculus. Scale bars = 500 μm (A, B, C), 10 μm (A1–A4, B1–B4, C1–C4), 50 μm (yellow inset in C).

For further examination of the CST, we defined four regions of interest to analyze bilateral components in the LF (Fig. 5A1, Fig.5 A2), and the VF (Fig. 5A3, Fig. 5A4), at the target region of the lesion. These were positioned to approximate the locations of the dlCST and vCST, but because of tissue changes after cavitation in the animals with SCI, the tract positions may have changed.

In the intact animal, the dCST identified by GFP^+^ and tdTomato^+^ labeling at T9–10 were located deep in the DF (Fig. 5A). At these levels in the animals with SCI, these tracts were not observed because of tissue loss from the central cavitation. There were few GFP^+^ and tdTomato^+^ axons in the LF (Fig. 5A1, Fig. 5A2), and VF (Fig. 5A3, Fig. 5A4) bins of intact animals. The bilateral components in LF and VF, however, were slightly more visible in vehicle-infused animals with SCI (Fig. 5B).

Both GFP^+^ and tdTomato^+^ axons of the LF and VF CST components on both sides of the spinal cord were more evident in the MSC-infused animals (Fig. 5C) than in the vehicle-infused animals (Fig. 5B). Note that the bilateral CST components in the LF in the MSC-infused animals (Fig. 5C1, Fig. 5C2) were more obvious than those in the vehicle-infused animals (Fig. 5B1, Fig. 5B2). In addition, both GFP^+^ and tdTomato^+^ CST axons were mixed bilaterally in the LF bins (Fig. 5C1, Fig. 5C2). In terms of the VF bins, both GFP^+^ and tdTomato^+^ VF axons in the MSC-infused animals (Fig. 5C3, 5C4) were more prominent than in the vehicle-infused animals (Fig. 5B3, 5B4).

Finally, the optical intensities of GFP^+^ and tdTomato^+^ bilaterally in the LF bins (Fig. 5C1, Fig. 5C2) were more prominent than those in the bilateral VF bins (Fig. 5C3, Fig. 5C4). The total abolishment of the dCST fibers in the central core of the lesion is shown in Fig. 5C5.

### Bridging communications between the dCST and axons in the LF just above and below the lesion core

**FIG. 6.**
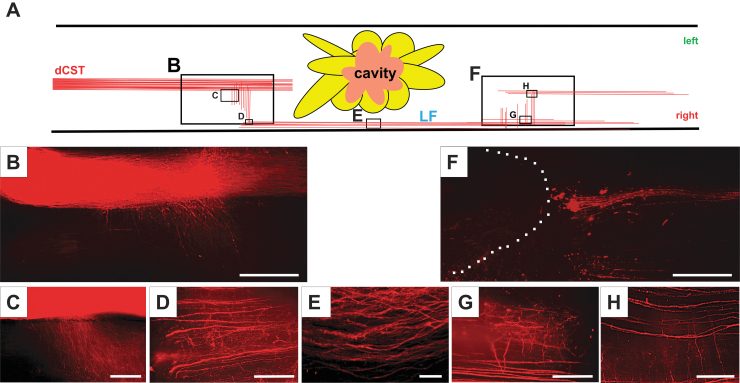
Bridging communications between dorsal corticospinal ract (dCST) and the axons in the lateral funiculus (LF) just above and below the lesion core were observed only in the mesenchymal stem call (MSC) treated group. (**A**) Schema of the spinal cord with cavitation showing the horizontal view of the spinal cord injected with adeno-associated viral vector-8-chicken beta-actin-tdTomato (AAV-8-CAG-tdTomato—red) into the left cortex. The light sheet fluorescence microscopy images with cleared spinal cord tissue: rostral to the lesion core (**B**) and LF (**E**) and caudal (**F**) to the lesion core. **C, D**, and **G, H** are higher magnifications of B and F, respectively. Scale bars = 1 mm (B, F), 200 μm (C), 100 μm (E), 50 μm (D, G, H).

To focus on the communication between the dCST and the axons in the LF in the MSC-infused animals with SCI, we used only tdTomato expressing AAV-8-CAG injected into the left cerebral hemisphere. We cleared the spinal cord tissue using a modified version of the advanced CUBIC protocol^31^ and examined the sections using LSFM (see Methods). The cartoon in Fig. 6A shows a horizontal view of the injured spinal cord with cavitation. Robust tdTomato^+^ dCST axons were observed just rostral to the lesion core (Fig. 6B), and labeled fine caliber axons projected into the LF (Fig. 6C).

Some of the fine axons formed radial projections relative to the longitudinally projecting axons in the LF (Fig. 6D). The axons in the LF running outside the lesion cavity are shown in Fig. 6E. Caudal to the lesion, some of these axons in the LF run vertically (Fig. 6G) to travel in the dCST caudal to the lesion (Fig. 6H). These axons, which converged to the dCST caudal to the lesion, are shown in Fig. 6F. No axons were observed within the lesion core (left side of the dashed line in Fig. 6F). LSFM imaging of optically cleared injured spinal tissue clearly demonstrated bridging connections between the dCST and the axons in the LF rostral to and just caudal to the lesion core. This phenomenon was only observed in MSC-treated animals, not in intact or vehicle-infused animals.

### Non-enhanced CSTs and non-bridging connections at areas distant from the lesion

We examined the dlCST and vCST axons away from the lesion core to address two questions. One is whether the existing well-known dlCST and vCST projecting from the cortex were enhanced in areas rostral to the lesion. The other is whether the bridging connections were formed immediately rostral and caudal to the lesion cores.

**FIG. 7.**
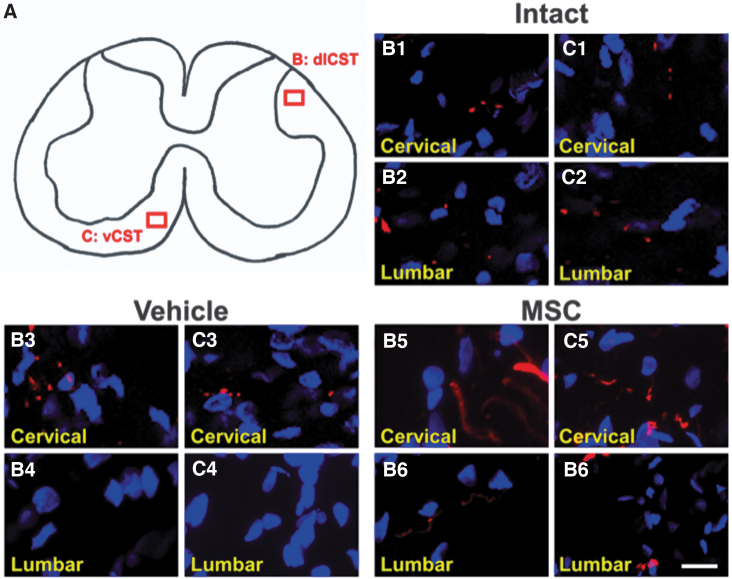
The dorsal corticospinal tract **(**dlCST) and ventral corticospinal tract (vCST) at areas away from the lesion. Diagram (**A**) illustrates dlCST and (**B**) illustrates vCST (**C**). Confocal images of the coronal spinal cord in intact, vehicle, and mesenchymal stem cell (MSC) groups injected with adeno-associated viral vector-8-chicken beta-actin-tdTomato (AAV-8-CAG-tdTomato—red) into the left cortex at regions away from the lesion core, including cervical level five and lumbar level two counterstained with 4',6-diamidino-2-phenylindole (DAPI—blue). Scale bar = 10 μm (B, C).

We examined dlCST and vCST away from the lesion core, including C5 (cervical) and L2 (lumbar) levels, in all groups. We found that there were no apparent enhancements of the dlCST and vCST at areas very proximal (C5 level) or distal (L2 level) to the lesions (Fig. 7). In terms of dlCST, there were tdTomato^+^ axons at the C5 level in all groups (intact; Fig. 7B1, vehicle; Fig. 7B3, MSC; Fig. 7B5); however, tdTomato^+^ axons were observed only in intact (Fig. 7B2) and MSC (Fig. 7B6) groups caudal to the lesion. Note the absence of tdTomato^+^ axons in the vehicle group (Fig. 7B4).

In terms of vCST, there were tdTomato^+^ axons at the C5 level in all groups (intact; Fig. 7C1, vehicle; Fig. 7C3, MSC; Fig.7 C5); however, tdTomato^+^ axons were observed only in intact (Fig. 7C2) and MSC groups (Fig. 7C6) caudal to the lesion. Note the absence of tdTomato^+^ axons in the vehicle group (Fig. 7C4). These observations suggest that existing dlCST and vCST projecting from the cerebral cortex are not enhanced at these areas far from the lesion sites, and the bridging connections between the dlCST and the axons in the LF become obvious both immediately rostral and immediately caudal to the lesion core after intravenous infusion of MSCs.

## Discussion

In this study, we demonstrated that the injured main component of the CST (dCST) rostral to the lesion core formed an enhanced axonal network that coursed parallel to the minor components of the CST after MSC treatment. Caudal to the lesion core, these axons appear to converge back to the cross-sectional position of the major component of the CST (dCST) in the deep DF. This unique phenomenon was evidenced by the enhanced imaging of axons in the CST pathways after MSC infusion.

The enhanced axonal network observed in this study in the minor components of the CST travel around the lesion core. Previous studies suggested that the minor components of the CST may be crucial for recovery by compensating for the loss of the main component (dCST) after damage of CST axons in the DF.^7^ These results suggest two possibilities: (1) new axons “sprouted” and regenerated from the injured dCST axons and joined the projections of the minor pathways, or (2) pre-existing very fine caliber axon collaterals became enhanced in size and therefore now more visible.

Several findings in this study support the hypothesis of either *de novo* axonal sprouting or enhancement of pre-existing fine caliber fibers after MSC treatment. First, while descending CST axons (GFP^+^ and tdTomato^+^ signals) are reduced in the caudal DF (dCST) after SCI, they were observed in the MSC group. Second, a mixture of GFP^+^ and tdTomato^+^ signals (CST axons from both left and right hemispheres) were observed in the LF and VF, but were more pronounced in the LF on both sides of the lesion core (Fig. 4 and Fig. 5) in the MSC group. This indicates that the axons in LF were enhanced (either in number or caliber to allow greater detection) after infused MSCs.

Third, we observed increased axonal communication between dCST and axons in the LF both just rostral and caudal to the lesion core (Fig. 6) in the MSC group, with convergence of axons to the dCST with appropriate laterality caudal to the lesion core. This suggests either axonal regeneration or visual enhancement of pre-existing descending CST axons.

Fourth, in regions more distant from the lesion core, both caudal (C5) and rostral (L2) (Fig. 7), tdTomato^+^ expression was not significantly enhanced in the dlCST or vCST on either side of the spinal cord in the vehicle- and MSC-infused groups. This indicates that existing dlCST and vCST axons projecting from the cerebral cortex are not enhanced at these areas far from the lesion sites. Rather, the bridging fine caliber connections between the dlCST and the axons in the LF become obvious both immediately rostral and immediately caudal to the lesion core after infused MSCs.

Thus, our data suggest that fine caliber connections in the dCST and the axons in the LF just above and below the lesion core were enhanced, as manifested by increased axonal diameter, and were more detectable after intravenous infusion of MSCs.

In the current study, we used a viral vector-based neuroanatomical, non-transsynaptic, axonal tracing method with GFP and tdTomato fluorescent-expressing AAVs to study the direct circuit reorganization. An advantage of this method is that it is non-transsynaptic, thus avoiding potential transsynaptic labeling of indirect propriospinal relay pathways. We used both confocal with frozen section and light-sheet fluorescence microscopy with an advanced optically cleared tissue method for whole spinal cord that allowed visualization of individual CST axonal fibers at various regions relative to the SCI lesion core. Future studies might be performed to elucidate the possible association of propriospinal neurons in the injured spinal cord as well.

From the data presented, we cannot determine whether the new components of the CST were from regenerated axons or whether pre-existing fine caliber axon collateral pathways from the dCST in LF were enhanced after infusion of MSCs. The latter possibility, however, seems the most likely because of the remarkable vertical convergence of the axons of the LF into the appropriate anatomical position of the dCST below the lesion.

The prospect of dCST axons regenerating from above the lesion and regenerating through a hostile growth environment rich in myelin inhibitory proteins and other inhibitory molecules of the lesion scar^32^ and targeting a small anatomically defined region seems highly improbable. It seems more likely that there is an enhancement (increased diameter) of low-detectable pre-existing fine caliber axons to allow greater visualization with the employed techniques than regeneration of axons that could rejoin the dCST after SCI.

It also should be noted that there is a time difference between the AAV tracing and the cavity formation in the spinal cord after SCI induction—i.e., it requires time for AAV to travel from the cortex to the spinal cord. We selected the timing of cortical AAV injection in this study to consider this time difference.

Cortical injected AAVs expressing GFP^+^ and tdTomato^+^ tracer did not arrive at T10 (the epicenter of SCI) for at least three weeks after AAV injection in an intact animal (Supplementary Fig. S1). It is well known that the central core of the SCI including dCST forms spinal cavitation four weeks after SCI induction using an IH impactor.^33^ The AAV was injected two weeks after SCI induction and reached the epicenter of SCI at about “five-weeks” after SCI. Because we injected AAV two weeks after SCI induction, AAV could not pass through the damaged central core of spinal tissue including the dorsal funiculus because the cavity would be formed at the time of arrival of AAV from the cortex five weeks after SCI induction (3 weeks after cortical AAV injection).

We confirmed the bright signals of both GFP and tdTomato in the dorsal funiculus indicating that the cortical injected AAV arrived at T10 six weeks after cortical AAV injection in an intact animal (Supplementary Fig. [B]). Therefore, the dCST axons just above the lesion, which were traced by AAV, had to form bridging communications between dCST and the axons in the LF just above and below the lesion core in the MSC-infused animals.

Finally, although the mixture of GFP^+^ and tdTomato^+^ AAV axons was visible on both sides of the DF in regions just caudal to the lesion core (Fig. 4C3-d), the converged GFP^+^ and tdTomato^+^ AAV axons were gradually positioned to their appropriate lateralization in the most distal sections (position 5) (Fig. 4C5-d). If the AAVs^+^ dCST axons could pass through the lesion core, GFP^+^ and tdTomato^+^ AAV axons would be positioned to their appropriate lateralization even in a region just caudal to the lesion core. Yet, the GFP^+^ and tdTomato^+^ AAV axons were mixed in a region just caudal to the lesion core in this model system. Therefore, we stress that the AAV^+^ dCST axons did not pass through the lesion core in this study.

Collectively, this phenomenon observed only in the MSC group might be a novel therapeutic mechanism for acute SCI after intravenous infusion of MSCs. Thus, these data shed light on a potential new therapeutic mechanism. This model suggests that fine caliber pre-existing axons not readily detectable with conventional tracing methods increase in diameter and contribute to improved conduction to motor neurons below the SCI site. Additional work will be needed to confirm this hypothesis.

This novel observation, however, suggests that it is possible that infused MSCs activate a compensatory system in the injured nervous system to use alternative functional routes by enhancing axonal diameter and conduction velocity and possibly synaptic reorganization. This is consistent with the concept that the underlying mechanism of MSCs is to provoke therapeutic efficacy in neural diseases by activating compensation systems in the central nervous syserm.^27,34^

In previous studies, we demonstrated that systemic infusion of MSCs not only impacted the injured neural tissue, but also the non-affected tissue. In a cerebral ischemia model, infused MSCs activated the compensatory system of the non-infarcted cortex, which was projected through interhemispheric cortical connections (corpus callosum) from the contralateral infarcted cortex.^27,34^ In a neonatal hypoxia-ischemic model, intravenous infusion of MSCs facilitated the growth of brain tissue in non-affected brain hemispheres.^35^

These studies indicate that infused MSCs may have effects on non-directly injured neural tissue, which may compensate for functional improvements. Taken together, it is conceivable that the interaction between the dCST and the axons in the LF observed in this study occurs as a therapeutic mechanism of infused MSCs in SCI. In addition, this further suggests that the intravenous cell delivery route might be a more appropriate way to allow MSCs to act not only on the injured lesion, but also on non-affected neural tissue to enhance functional improvement.

## Conclusions

There is an enhancement of observable projections between the dCST and axons in the LF both rostral and caudal to the SCI lesion core after intravenous infusion of MSCs. This was demonstrated using a combination of AAV anterograde, non-transsynaptic, axonal tracers and advanced tissue clearing techniques. The results suggest either axonal regeneration from the dCST or axonal enhancement—i.e., an increased axonal diameter of low-detectable pre-existing fine caliber axons to allow greater detection. This latter prospect is more likely than regeneration or sprouting of dCST axons because of the unlikely possibility that regenerated axons could travel through a regeneratively hostile pathological environment and precisely rejoin the dCST caudal to the lesion.

Clearly, more work is necessary to study this possibility, but the prospect of enhancement in axonal diameter of pre-existing small caliber axons by MSC delivery has important implications for MSC-induced functional recovery in SCI.

## Supplementary Material

Supplemental data
