# Supplementary material for: Enhanced Network in Corticospinal Tracts after Infused Mesenchymal Stem Cells in Spinal Cord Injury
Source: J Neurotrauma. 2022 Nov 30;39(23-24):1665–77. doi: 10.1089/neu.2022.0106 (PMC9734021; doi:10.1089/neu.2022.0106)
Supplement: Supplemental data [file Supp_FigS1.docx]

**Supplementary Figure**

**
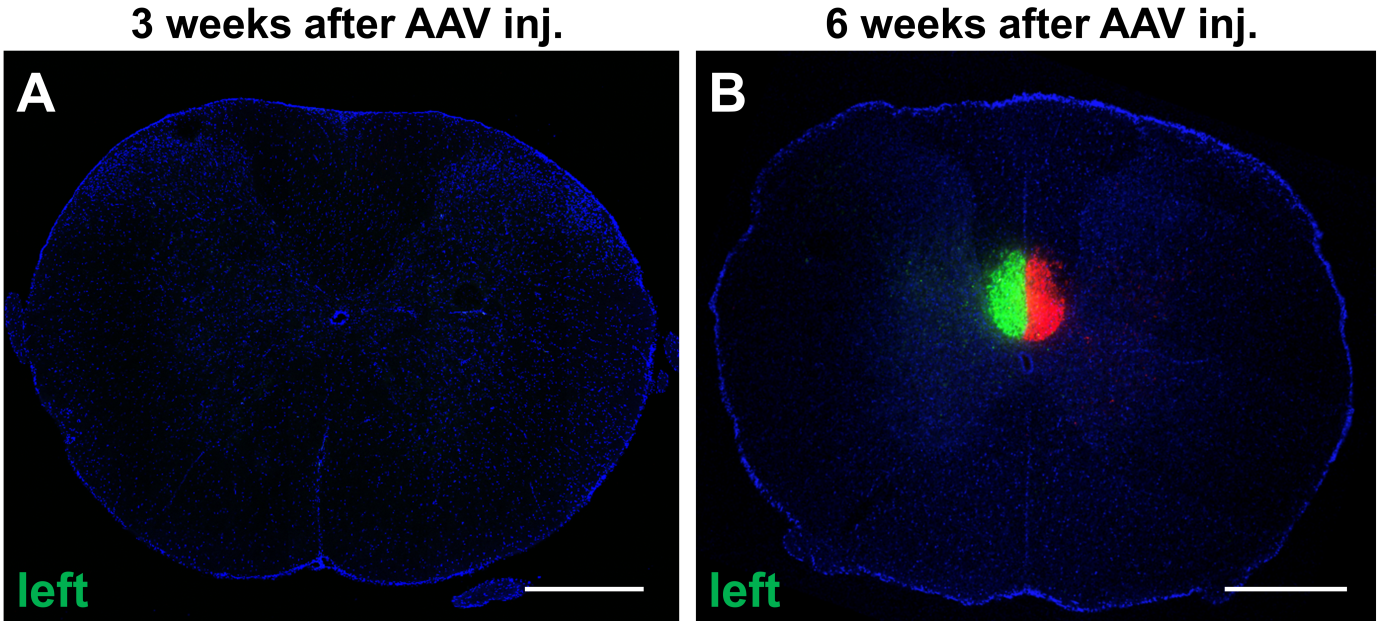
**

Coronal spinal cord sections at T10, 3 (A) and 6 weeks (B) after injection of AAV-8-CAG-GFP (green) virus into the right cortex and AAV-8-CAG-tdTomato (red) into the left cortex in an intact rat. T10 level is the targeted epicenter in SCI rats. Scale bar = 300 μm (A, B).
